# Levels of Stress in Volleyball Referees During Official Matches—The Influence of the Referee Role and Level of Competition

**DOI:** 10.3390/sports12120319

**Published:** 2024-11-26

**Authors:** Zoran Nikolovski, Dario Vrdoljak, Nikola Foretić, Mia Perić, Vladimir Pavlinović, Ratko Perić, Vuk Karanović

**Affiliations:** 1 Faculty of Kinesiology, University of Split, 21000 Split, Croatia; zoran.nikolovski@kifst.eu (Z.N.); nikola.foretic@kifst.eu (N.F.); mia.peric@kifst.eu (M.P.); vladimir.pavlinovic@kifst.eu (V.P.); 2 High Performance Sport Center, Croatian Olympic Committee, 10000 Zagreb, Croatia; 3 Confédération Européenne de Volleyball, Grand Duchy of Luxembourg, 1940 Luxembourg, Luxembourg; vuk.karanovic@cev.eu; 4 Department for Exercise Physiology, Orthopedic Clinic Orthosport, 78000 Banja Luka, Bosnia and Herzegovina; ratkoperic@yahoo.com

**Keywords:** cortisol, alpha-amylase, hormonal system, nervous system, competition, volleyball referees

## Abstract

Volleyball referees, as athletes and staff members, are exposed to different stress levels which can be determined by measuring pre- and post-match levels of salivary cortisol (C) and alpha-amylase (AA). This study aimed to determine the dynamics of stress biomarkers in referees during official volleyball matches and the connection to the roles or level of competition. The participants in this study were nine international volleyball referees (three females and six males) with a mean chronological age of 48.23 ± 2.31 years. In this study, saliva samples were collected during 24 official matches during the European championship for senior women’s teams (Eurovolley 2021). The AA activity and C concentrations were determined from saliva samples. When the referees’ roles were assessed in line with their duties, the first referees’ salivary C levels showed a significant increase between the pre- and post-match measurements (*p* = 0.01), while in the second referees remained low. The reserve and challenge referees demonstrated a significant drop in their C concentrations (*p* = 0.00 and *p* = 0.02, respectively). Additionally, when assessing AA which accounts for the responsibilities of referees and the intensity of competition, the first (*p* = 0.06 and *p* = 0.07) and second referees (*p* = 0.01 and *p* = 0.00) showed an increase between the pre- and post-match measurements, respectively. At the same time, the AA activity did not show any significant change concerning the reserve and challenge referees. Our results indicate that referees’ roles and the level of competition may cause higher responses in “active referee roles”—mainly the first and second referees—while reserve and challenge referees showed no increase or even a decrease in the measured biomarkers. The observed changes in the stress markers can be explained by psychological or emotional effects and are dependent on the level of competition and the role referees are fulfilling.

## 1. Introduction

Stress is defined as a condition where a person under any uncontrollable challenge enters a state of arousal or anxiety [1]. The response to these challenges is fully individual and depends on the person’s capacity to resist different tasks [1]. In situations perceived by a person as strongly aversive and uncontrollable, whether physical or psychological, the levels of neurochemical stress markers will increase, and these can be measured in different bodily fluids [2].

The most examined biomarkers of stress are cortisol and alpha-amylase [3,4]. Cortisol (C) is involved in the stress response, and its concentrations represent the activity of the hypothalamic-pituitary-adrenocortical axis [5]. It is often used as a biomarker of physical and psychological stress [6]. Alpha-amylase (AA) catalyzes the hydrolysis of starch into smaller carbohydrate molecules, such as maltose [7]. Additionally, AA reflects the activity of the sympathetic nervous system. The levels of AA are highly related to an increase in noradrenalin, and as was reported, both AA and noradrenalin reflect an individual’s state of arousal [8]. According to recent studies, professionals that engage in competitive sport activities face the most stressful environments [9,10].

Previous studies [11,12,13] used enzyme AA and hormone C tests to assess how competition and competitive stress affect athletes. In team sports, pre- to post-game C level increases have been reported [12,14,15]. Additionally, C level increases have been also demonstrated in different individual sports (e.g., martial arts and tennis) when pre- and post-competition measurements were compared [13,16]. AA also showed a significant increase due to the amount of physical activity, being studied previously in a variety of sports [17,18]. AA has been found to be responsive to a variety of challenging situations, such as heat, environment, and cognitively oriented social or laboratory tasks [8,19,20,21].

In addition to players, coaches and staff members can also experience some level of competitive stress. As a consequence of prolonged stress, the complex and stressful environment in which trainers work can induce fatigue, frustration, or apathy [22,23]. In addition to athletes and coaches, other competition participants, such as referees, can endure stress as well. Previous studies explored which stressors are most common among referees [24,25]. Poor decision making and time limits were identified as powerful internal stressors [24]. On the other hand, the most frequent external stressors were fear of physical danger and verbal abuse from coaches or the audience [25]. Stressors can arise before, during, and after competitions. To assess the stressors present in all levels of competition, the main tool of examination in studies was questionnaires, whereas other more precise and objective methods are also available. Such methods include measurement of hormonal activity, where different stressors may activate the secretion of hormones and enzymes. A recent systematic review showed a lack of studies which address stress in sports referees during official competition using stress biomarkers and if different levels of competition influence the psychophysiological reactions of referees [26].

Volleyball is a dynamic sport which underwent numerous changes in recent years, and as such, the number and roles of referees were also modified accordingly. First and second referees are mainly responsible for rule application and match conduction from the beginning until the end. The reserve referee serves as security in case it is necessary to substitute either the first or second referee. The challenge referee’s role is that of a technology assistant for disputed situations where coaches can “challenge” referees’ decisions, with the number of such challenges being limited during one set.

Due to such diversity of roles, we speculated that different roles and activities might elicit different levels of stress in referees, and these differences can be attributed to their different tasks. In addition, we speculated that the level of competition might affect the level of stress of referees.

Therefore, this study aimed to determine the dynamics of stress biomarkers (C and AA) in referees during official volleyball matches and if their possible changes have a connection to the referees’ roles or level of competition.

## 2. Materials and Methods

### 2.1. Participants

The participants in this study were 9 international volleyball referees (3 females and 6 males) with a mean chronological age of 48.23 ± 2.31 years.

As the influence of gender on the levels of stress biomarkers is a known topic, and it was previously defined that this does not play an important role, we analyzed the results of different genders among the referees as a single group. The referees in this study were of an elite level and chosen by the European Volleyball Federation (CEV). This study was carried out during the European volleyball championship for senior women (Eurovolley 2021) in two pools (out of four pools). The selected referees were the best referees selected for the competition, depending not only on their quality, experience, and previous participation in the highest competitions but also by the neutrality of the referees, based on their nationality and the participating teams. All referees taking part in this study volunteered and were informed about the purpose of the study. Experimental procedures were completed following the Declaration of Helsinki, and they were approved by the corresponding authors’ institutional research ethics board (Ethics Board Approval No. 2181-205-02-05-23-024).

### 2.2. Procedure

In this study, saliva samples were collected throughout 24 official matches (group phase and final phase). The alpha-amylase activity and cortisol concentrations were determined from the saliva samples. All matches were played in the afternoon hours between 5:00 p.m. and 10:00 p.m. in two sports halls with an air condition-controlled environment (temperature = 22 °C; humidity = 35%). The referees’ roles alternated between matches, and all of them were in the position of either first, second, reserve, or challenge referee. During this competition, there were only two matches per day, and special attention was paid to a single referee in one day not having more than one “active role” (only one match a day) and the second referee rarely having an additional role (usually reserve referee) in the second match. Aside from this, the first referee for any day never had more than one match a day and never switched the roles.

### 2.3. Sampling and Handling

**Figure 1:**
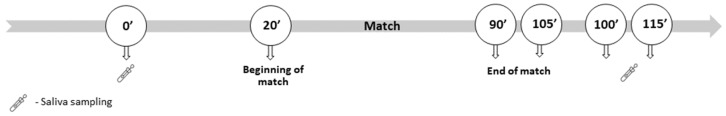
Sample collection timeline 30 min before a match and directly after a match (time of second sample collection is not standard because of the impossibility of always taking it at the same time due to different match durations).

Saliva samples were collected (see Figure 1) for salivary hormonal biomarker determination. Ten minutes before pre-match sample collection, the referees rinsed their mouths thoroughly with water. This sample collection took place at least 60 min after a major meal. Upon match completion, 10 min before each sample was collected, the referees rinsed their mouths again. For this purpose, SalivaBio Oral Swabs (SOSs) (Salimetrics LLC, State College, PA, USA) were used, being placed at the floor of the mouth under the tongue for 2 min. After collection, the swabs were placed into a storage tube and immediately placed on ice. Within 2 h following the sampling, the samples were frozen at −20 °C for later determination. On the day of analysis, the samples were thawed completely and centrifuged at 1500× *g* (3000 rpm) for 15 min. After centrifugation, assays were performed. Saliva cortisol was analyzed with a commercially available enzyme-linked immunosorbent assay (ELISA) purchased from Salimetrics LLC (State College, PA, USA) on a microplate reader (Infinite 200PRO, Tecan, Mannendorf, Switzerland). Standard curves were constructed according to the manufacturer’s instructions and commercially available standards, and quality control samples were used for all assays (Salimetrics LLC). The assay sensitivity for salivary cortisol was 0.007 µg/dL, with an average intra-assay coefficient of variance (CV) of 4.5%. All samples were analyzed in duplicate and in the same batch to avoid intra-assay variability. The samples of alpha-amylase were analyzed using a kinetic enzyme assay kit from the same supplier (Salimetrics LLC, State College, PA, USA). The average intra-assay CV was 5.5%. Values are expressed as the alpha-amylase concentration (U/mL). Salivary hormone concentrations were corrected for the salivary flow rate. All data were log transformed to reduce the non-uniformity of error, and normality was tested using the Kolmogorov–Smirnov test procedure. The Levene test checked for homoscedasticity.

### 2.4. Statistical Analysis

To determine the differences between referee roles, the Sign test was used. Furthermore, a multiple comparison ranks test was performed to determine the possible differences between the competition levels in all groups of participants. The *p* values in all analyses were tested with the magnitude-based Cohen’s effect size (ES) statistic with modified qualitative descriptors (trivial ES < 0.2; small ES = 0.21–0.60; moderate ES = 0.61–1.20; large ES = 1.21–1.99; and extremely large ES > 2.0).

The software Statistica ver. 13.0 (Dell Inc., Tulsa, OK, USA) was used for all analyses, and a *p* level of 95% (*p* < 0.05) was applied.

## 3. Results

The first referees’ levels of saliva C showed a significant increase between the pre- and post-match measurements (pre = 2.17 ± 1.33 µg/dL; post = 4.27 ± 3.39 µg/dL; *p* = 0.01). Meanwhile, the reserve (pre = 2.02 ± 0.90 µg/dL; post = 1.21 ± 0.89 µg/dL; *p* < 0.00) and challenge referees (pre = 1.56 ± 0.79 µg/dL; post = 0.99 ± 0.40 µg/dL; *p* < 0.00) experienced a drop in C concentrations between the pre- and post-match measurements.

Furthermore, moderate effect sizes were found for the first (0.82), reserve (0.91), and challenge referees (0.91) for the pre- and post-match measurements. Simultaneously, trivial or small effect sizes were detected in the second referees and when all referees’ roles were examined together (see Table 1).

There were statistically significant differences in salivary AA between the pre- and post-match measurements among all referees across all roles and when the first and second referees were observed separately (Table 2). The Sign test for all participants showed a significant increase in the levels of AA (pre = 81.17 ± 72.02; post = 100.43 ± 92.69; *p* = 0.00) between the pre- and post-match measurements. Additionally, the second referees’ (pre = 106.60 ± 95.55; post = 133.09 ± 122.31; *p* = 0.01) measurements showed a statistically significant increase in AA between the pre- and post-match measurements, while the measurements of AA for the first referees (pre = 88.92 ± 87.64; post = 140.10 ± 113.73; *p* = 0.06) showed an increase between the pre- and post-match measurements without reaching a level of significance.

Furthermore, small effect sizes were found among all referee roles (across all referees (0.23) and first (0.50), second (0.25), reserve (0.24), and challenge referees (0.30)).

No statistically significant differences existed between the group phase and final phase measurements (both pre-and post-match) for the first, second, reserve, and challenge referees. However, the second referees’ levels of saliva C showed a moderate effect size between the group phase (1.78 ± 0.55) and the final phase (2.30 ± 0.96) in the pre-match measurement (0.70). Meanwhile, the reserve referees demonstrated similar levels of C in both phases of competition for the pre-match measurements (group phase = 1.94 ± 1.06 µg/dL; final phase = 2.14 ± 0.66 µg/dL; ES = 0.22) and post-match measurements (group phase = 1.18 ± 1.12 µg/dL; final phase = 1.26 ± 0.46 µg/dL; ES = 0.09). A similar trend was observed for the challenge referees in the pre-match measurements (group phase = 1.59 ± 0.80 µg/dL; final phase = 1.51 ± 0.83 µg/dL; ES = 0.10) and post-match measurements (group phase = 1.00 ± 0.45 µg/dL; final phase = 0.96 ± 0.33 µg/dL; ES = 0.10). The results for the first referees demonstrate higher levels of salivary C compared with the other referees. However, there were no significant differences between both phases of competition for the pre-match measurements (group phase = 2.10 ± 1.30 µg/dL; final phase = 2.29 ± 1.46 µg/dL; ES = 0.14) and post-match measurements (group phase = 3.85 ± 2.62 µg/dL; final phase = 5.02 ± 4.55 µg/dL; ES = 0.33) (see Table 3).

There were statistically significant differences between the group phase and final phase measurements when all referees were observed as a single group and when the second referees were observed separately. The Sign test for all participants showed a significant difference in the levels of AA between the group phase (74.45 ± 46.37) and the final phase (141.99 ± 127.95) in the post-match measurements (*p* < 0.00). Additionally, the second referees’ measurements showed a similar trend in AA between the competition levels for both the pre- match measurements (group phase = 59.02 ± 31.67 µg/dL; final phase = 180.61 ± 115.55 µg/dL; *p* < 0.00) and post-match measurements (group phase = 76.78 ± 43.26 µg/dL; final phase = 222.13 ± 153.72 µg/dL; *p* < 0.00).

Furthermore, small effect sizes were found among all referee roles (0.66) and the first referees (0.67) in the pre-match measurements. For the post-match measurements, all referee roles (0.78) and the first (0.91) and challenge referee (0.78) roles demonstrated moderate effect sizes. The largest effect sizes were observed for the second referee in both the pre-match measurements (1.56) and post-match measurements (1.40) when comparing different levels of competition (see Table 4).

## 4. Discussion

This study aimed to determine the differences in C and AA dynamics in volleyball referees. Our study was carried out during the European volleyball championship for senior women, comparing different levels of competition (namely the group and final phases) and different referee roles. Our data supported a number of significant conclusions. (i) Significant differences were found in the AA and C levels when comparing the various roles of the referees. (ii) When comparing referee groups based on competition levels, variations in AA (but not in levels of C) were found. (iii) Possible pre-competitive anxiety was observed. This was indicated by the existence of moderate-to-high ESs in the pre-match C and AA values of the group and final phases of competition when the first and second referees were compared.

All referees in this competition were referees of the highest level—A list—of the European Volleyball Federation (CEV), and most of them were also included for the matches organized by the International Volleyball Federation (FIVB), which is responsible for the matches in the Olympic Games and Nations League, the highest world competitions.

Assignment of the referees to their specific roles in the matches is dependent on numerous criteria. First, all of them have to whistle. Second, distribution of the national teams and referees’ nationalities has to be taken into consideration for the reason of neutrality. Sometimes, due to disputes in previous matches, some referees are asked to be excluded from refereeing particular teams.

The potential influence of psychological factors, such as decision-making responsibility, role visibility, or experience with handling stressful situations, might be real reasons to have different stress responses between “active role” (first and second) referees and the rest of the referees (reserve and challenge) with less “active” roles, as was observed in the analyses of the results.

Previously, C was shown to be a reliable biomarker of physical and mental stress [5,27,28]. To be precise, C reflects activation of the hypothalamic-pituitary-adrenocortical axis, which is involved in physiological stress responses [6,29]. In our study, we evaluated the endocrine response of C pre- and post-match in volleyball referees and its dependence on the referee’s role and competition level. Pre- and post-match C level differences were noted, with the first referees differing considerably from the second, reserve, and challenge referees while the second referees differed from both the reserve and challenge referees. These results indicate that “active referee” roles (first and second referees) experience different endocrine system responses compared with the other “less active” referee roles. Moreover, the C values did not show significant differences among the referees’ roles when the competition level was studied (group phase versus final phase). Even though we might presuppose that in the final phase of competition, stress levels should be higher due to the higher standards and expectations of performance, and this might produce higher levels of C, our data did not completely corroborate such speculation. Anyhow, C showed higher values only for the second referees in the pre-match measurements (even though the ESs in the first and second referees were small and moderate, respectively). This finding might be considered for future analysis. Some of the explanations for this might include circadian variability (matches were played in the afternoon) or the durations of the matches. What we observed was that when considering an intermediate sample of saliva taken after the second set, or about 60 min after the first sample), C levels rose significantly but decreased toward the end of the match. These findings will require further analysis introducing more time points in order to better understand the dynamics of stress biomarkers during a volleyball match.

In a study by Nogueira, Fontes et al. [30], football referees reported negative emotions related to the perception of performance and connected these negative emotions to stressors which they had been subjected to during matches. Kokaly, Peñailillo et al. [26] reported that C concentrations in football referees increased by 48.8% after a match. They speculated that this increase was due to the physical and mental demands of football. Hence, differences between referee roles could be explained by the difficulty of the work they perform and the psychological and mental strains they are subjected to, as the physical demands during a volleyball match are not comparable to those of their football counterparts (reserve and challenge referees sit, while first referees mostly stand during the match and second referees stand and walk minimally). While C liberation reflects activation of the hypothalamic-pituitary-adrenocortical axis, the production of AA is regulated by the autonomic nervous system. Liberation of catecholamines, induced by both exercise and psychological stress, correlates with an increase in AA levels [27,28].

When the mean values of AA are compared, it can be noted that first and second referees had a higher increase in AA between the pre- and post-match levels than the reserve or challenge referees. It is interesting to examine the challenge referees which separates them from other referees. The challenge referee still has a relatively important responsibility with a possible stressful role, which was also observed in the form of an increase in AA between the pre- and post-match measurements (without arriving at the significance level). The analysis of the results in the pre-match and post-match measurements between the group phase and final phase of the competition showed a significant difference in the levels of salivary AA. This difference was observed in the samples of the first and second referees. To be precise, the levels of salivary AA during the final phase were higher than those during the group phase for both referee roles. Anyhow, a trend without statistical significance was observed among the first referees, while a statistically important difference was detected only among the second referees. Additionally, in the saliva samples of the challenge referees, we detected higher values during the final phase but only in the post-match measurement, while the reserve referees demonstrated similar values in both the group phase and final phase matches.

The AA results show that the higher the level of competition, the more powerful and apparent activation of the nervous system was. The post-match measurements were also higher in the final phase of competition. Higher post-match values could indicate that refereeing at this level is more stressful for these referees. Similarly, the challenge referees were experiencing stress during the match, even though their pre-match AA levels were similar when comparing the group and final phase matches. This trend in arousal, anxiety, and stress was not observed in the reserve referees, who showed the same values during the group and final phases.

Numerous previous studies reported a rise in AA levels during competitive sports and other physical activities [6,31,32]. These investigations concentrated on performance-related stress or the conditions that athletes face while competing. However, volleyball referees do not have significant physical stress during the course of a game. Nevertheless, an increase in the aforementioned biomarker was apparent. Therefore, it can be concluded that other external and internal factors influence these responses (e.g., audience, players, and coaches). These data validate the results, which show that the referees’ neurological systems and their psychological and emotional responses to the demands of the game were amplified. Analysis of the results demonstrates that the first and second referees were exposed more to internal (e.g., bad decision making or time pressure) then external stressors (e.g., verbal abuse from the coaches or audience or fear of physical harm) [24,25]. In addition to referee roles, the level of competition is significant and plays a role in inducing a stronger stress response. Their biomarkers (C and AA) rose at a faster rate than those among the referees in the other two positions. Furthermore, an AA increase was noticed in all referee roles, whereas a C increase was present only in the roles of first and second referee. Such a pattern is expected and may be explained by the nature of the nervous system, which is triggered much faster than the endocrine system. First and second referees in volleyball are more exposed to the audience, players, and coaches and are the subject of undivided attention. Their decisions affect both the teams and their supporters. Our findings show that they are more exposed to stressors during the course of a match compared with the reserve or challenge referees, and the stress response is also dependent on the competition level, with a higher level inducing more stressful reactions. First and second referees are “visible heads” and exposed to the pressure of the audience. They are exposed to the possible protests of their decisions from the side of the players and coaches based on their decision making. Also, they are monitored by the referee coaches; their decisions, position changes, dispute handling, match dynamics handling, treatment of participants in the match (including officials), and their evaluation may affect their rankings during a competition and their future assignments. Even though most of them are experienced with many matches whistled, all of the above-mentioned factors can affect their state of arousal or anxiety before or during matches (some of them actual factors and some anticipatory).

Another effect which might be associated with the competition level is the “pre-competition anxiety” effect. It is important to mention that our investigation identified variations in the levels of AA activity and concentrations of C before matches. Among all referees collectively, both the first and second referees exhibited a moderate-to-high effect size (ES) for the AA values, whereas the second referees showed a high ES in the C levels. This increase observed between two different levels of competition might suggest that the competition level could elicit an endocrine response before a match, followed by an increase in the levels of C and AA.

One work by Dehghan, Khodaei [33] reported that the cortisol and AA concentrations of an elite group of inline skaters significantly increased 1 h before competition compared with on a rest day. The authors concluded that the optimum increase in adrenal and SNS activity occurs before a competition, which can improve athletic performance.

In another study with athletes of archery, the author reported that competitive anxiety in archery increases sAA and sC levels from immediately before a competition until after the end of a competition but not 30 min before a competition [34].

### Limitations of This Study

This study has several limitations.

The number of referees was small, but the number of referees in one European championship is limited due to various limitations imposed by the European Volleyball Federation and host country federation. Therefore, we could not increase the number of referees, but we collected data from 24 official matches, which means that we had analyses of four different referee roles in 24 different matches.

Additionally, it was not possible to conduct baseline measurements of the biomarkers. Referees usually come to competitions from their respective countries one day before the competition starts. This day is full of activities for them (e.g., medical checkups and referee clinics) both theoretical and practical, including verification of the competition court and familiarization with equipment. These activities create stressful situations and cannot represent valid baseline measurements.

We were not able to assess and correlate the levels of the stress biomarkers and the referees’ performance.

Additionally, we were not in a position to continuously monitor their heart rates, which might be useful tool for assessment of the autonomous nervous system. We were not allowed to place HR monitors on the referees as they were already wearing headphones and microphones and holding tablets. Thus, we were discouraged from placing additional wearables on them in order not to disrupt their concentration.

Future studies are encouraged to introduce this approach.

## 5. Conclusions

Volleyball referees are exposed to different levels of stress, which can be demonstrated by measuring the pre- and post-match levels of salivary C and AA. The differences observed indicate that the referees’ roles may influence higher responses in “active referee roles”—mainly the first and second referees—while the reserve and challenge referees showed no increase or even a decrease in the measured biomarkers. Also, with higher levels of competition, referees experience higher levels of stress before and during competition. Keeping in mind that volleyball referees are not exposed to physical effort throughout a match, the observed changes in the stress markers can be explained by psychological and emotional effects and are dependent on the roles referees are fulfilling. Anticipatory stress observed through increased levels of AA and C before and during a competition is associated with the level of competition, which might suggest that referees should be instructed how to properly “relax” before a competition.

### Practical Recommendations

We would suggest that referees use slow-paced breathing at low frequencies as a method of relaxation prior to matches. Research works have published one case study on using slow-paced breathing and additionally carried out a study based on slow-paced breathing in one athlete’s academy with adolescents. Positive outcomes were observed when applying the mentioned procedure). The last study was carried out with the assistance of a certified sport psychologist.

The authors of this study are preparing results for publication from a second study with volleyball referees, where we will address the influence of relaxation techniques such as slow-paced breathing at low frequencies in match preparation.

Due to the fact that we are in the extremely early stages of this study (we performed the relaxation techniques before the matches, collected samples, and performed questionnaires of the level of anxiety and heart rate monitoring), we are not in a position to share these insights. We expect that we will soon analyze all data and prepare them for publication.

## Figures and Tables

**Table 1:** Descriptive statistics for measured salivary C, with differences obtained by Sign test analysis and effect size (ES).

Variable	AM	SD	Z	*p* Value	ES	95% CI
	All Groups					
C_pre	1.94	0.98	1.26	0.21	0.17	−0.24–0.58
C_post	2.24	2.33				
	1st Referee					
C_pre	2.17	1.33	2.77	0.01 *	0.82 ^¥^	−0.06–1.69
C_post	4.27	3.39				
	2nd Referee					
C_pre	1.99	0.76	0.00	1.00	0.43	−0.40–1.26
C_post	2.58	1.79				
	Reserve referee					
C_pre	2.02	0.90	3.06	0.00 *	0.91 ^¥^	0.07–1.75.
C_pre	1.21	0.89				
	Challenge referee					
C_pre	1.56	0.79	2.35	0.02 *	0.91 ^¥^	0.03–1.79
C_post	0.99	0.40				

C = cortisol; AM = arithmetic mean; SD = standard deviation; Z = test value; * = *p* level of significance, with statistical significance of *p* < 0.05; ES = effect size, with significant differences between groups indicated by ^¥^.

**Table 2:** Descriptive statistics for measured salivary AA, with differences obtained by Sign test analysis and effect size (ES).

Variable	AM	SD	Z	*p* Value	ES	95% CI
	All Groups					
AA_pre	81.17	72.02	3.14	0.00 *	0.23	−0.18–0.64
AA_post	100.43	92.69				
	1st Referee					
AA_pre	88.92	87.64	1.92	0.06	0.50	−0.35–1.35
AA_post	140.10	113.73				
	2nd Referee					
AA_pre	106.60	95.55	2.50	0.01 *	0.25	−0.57–1.07
AA_post	133.66	122.31				
	Reserve Referee					
AA_pre	69.61	46.03	−0.20	0.84	0.24	−0.57–1.04
AA_post	60.06	33.19				
	Challenge Referee					
AA_pre	59.46	35.29	1.49	0.14	0.30	−0.54–1.14
AA_post	70.06	36.33				

AA = alpha-amylase; AM = arithmetic mean; SD = standard deviation; Z = test value; * = *p* level of significance, with statistical significance at *p* < 0.05; ES = effect size.

**Table 3:** Descriptive statistics for measured salivary C with differences, obtained by Mann–Whitney test analysis and effect size (ES).

Variable	Final Phase			Group Phase			Z	*p*	ES	IC 95%
	N	Mean	SD	N	Mean	SD				
All Groups										
C_pre	35	2.07	1.01	56	1.85	0.96	1.09	0.28	0.23	−0.20–0.65
C_post	35	2.39	2.68	56	2.15	2.09	0.60	0.55	0.10	−0.32–0.53
1st Referee										
C_pre	8	2.29	1.46	14	2.10	1.30	−0.10	0.92	0.14	−0.67–0.95
C_post	8	5.02	4.55	14	3.85	2.62	0.31	0.76	0.33	−0.49–1.15
2nd Referee										
C_pre	9	2.30	0.96	14	1.78	0.55	1.35	0.18	0.70 ^¥^	−0.14–1.53
C_post	9	2.59	1.25	14	2.58	2.11	0.66	0.51	0.01	−0.81–0.82
Reserve Referee										
C_pre	10	2.14	0.66	14	1.94	1.06	0.79	0.43	0.22	−0.60–1.03
C_post	10	1.26	0.46	14	1.18	1.12	1.14	0.25	0.09	−0.72–0.90
Challenge Referee										
C_pre	8	1.51	0.83	14	1.59	0.80	−0.10	0.92	0.10	−0.77–0.97
C_post	8	0.96	0.33	14	1.00	0.45	−0.24	0.81	0.10	−0.77–0.97

C = cortisol; SD = standard deviation; Z = test value; ES = effect size, with significant differences between groups indicated with ^¥^.

**Table 4:** Descriptive statistics for measured salivary AA, with differences obtained by Mann–Whitney test analysis and effect size (ES).

Variable	Final Phase			Group Phase			Z	*p*	ES	IC 95%
	N	Mean	SD	N	Mean	SD				
All Groups										
AA_pre	35	109.01	103.13	56	63.77	33.33	1.90	0.06	0.66 ^¥^	0.22–1.09
AA_post	35	141.99	127.95	56	74.45	46.37	3.19	0.00 *	0.78 ^¥^	0.34–1.21
1st Referee										
AA_pre	8	127.71	136.91	14	66.75	29.31	1.19	0.23	0.67 ^¥^	−0.16–1.51
AA_post	8	204.10	154.10	14	103.52	64.39	1.81	0.07	0.91 ^¥^	0.06–1.76
2nd Referee										
AA_pre	9	180.61	115.55	14	59.02	31.67	3.50	0.00 *	1.56 ^¥^	0.64–2.49
AA_post	9	222.13	153.72	14	76.78	43.26	3.37	0.00 *	1.40 ^¥^	0.50–2.30
Reserve Referee										
AA_pre	10	65.75	59.95	14	72.37	35.22	−1.55	0.12	0.14	−0.67–0.95
AA_post	10	64.00	41.09	14	57.24	27.56	0.03	0.98	0.20	−0.61–1.01
Challenge Referee										
AA_pre	8	63.84	32.24	14	56.95	37.87	0.72	0.47	0.19	−0.68–1.06
AA_post	8	87.22	42.61	14	60.26	29.48	1.60	0.11	0.78 ^¥^	−0.12–1.68

AA = alpha-amylase; SD = standard deviation; Z = test value; * = *p* level of significance, with statistical significance at *p* < 0.05; ES = effect size, with significant differences between groups indicated with ^¥^.

## Data Availability

All data are included in the manuscript’s body.
